# Acute effects of a chewable beetroot-based supplement on cognitive performance: a double-blind randomized placebo-controlled crossover clinical trial

**DOI:** 10.1007/s00394-023-03265-y

**Published:** 2023-10-24

**Authors:** Maria Grazia Vaccaro, Bernardo Innocenti, Erika Cione, Luca Gallelli, Giovambattista De Sarro, Diego A. Bonilla, Roberto Cannataro

**Affiliations:** 1 Department of Medical and Surgical Sciences, University of Magna Graecia, Catanzaro, Italy; 2 BEAMS Department, Universitè Libre de Bruxelles, Brussels, Belgium; 3 Department of Pharmacy, Health and Nutritional Sciences, University of Calabria, Rende, Italy; 4 Galascreen Laboratories, University of Calabria, Rende, Italy; 5 Clinical Pharmacology and Pharmacovigilance Operative Unit, Department of Health Science, University of Magna Graecia, Mater Domini Hospital, Catanzaro, Italy; 6 Research Division, Dynamical Business and Science Society–DBSS International SAS, 110861 Bogotá, Colombia; 7 Research Group in Physical Activity, Sports and Health Sciences (GICAFS), Universidad de Córdoba, 230002 Montería, Colombia; 8 Research Group in Biochemistry and Molecular Biology, Universidad Distrital Francisco José de Caldas, 110311 Bogotá, Colombia

**Keywords:** Nitric oxide, Nitrates, Dietary supplements, Nitric oxide, Neuropsychological tests, Cognitive function

## Abstract

**Background:**

Dietary nitrate (NO_3_^−^) has been shown to be useful as an ergogenic aid with potential applications in health and disease (e.g., blood pressure control). However, there is no consensus about the effects of dietary NO_3_^−^ or beetroot (BR) juice supplementation on cognitive function.

**Objective:**

The aim of this study was to evaluate the effects of a single dose of a chewable BR-based supplement on cognitive performance.

**Methods:**

A double-blind randomized placebo-controlled two-period crossover clinical trial was carried out based on the extension of the CONSORT guidelines for randomized crossover trials. A total of 44 participants (24 F; 20 M; 32.7 [12.5] years; 66.3 [9.0] kg; 170 [9.2] cm; 22.8 [1.4] kg/m^2^) were randomly allocated to receive first either four BR-based chewable tablets (BR-CT) containing 3 g of a *Beta vulgaris* extract (RedNite^®^) or four tablets of a placebo (maltodextrin). A 4-day washout period was used before crossover. Ninety minutes after ingestion of the treatments, a neuropsychological testing battery was administered in each period. The trial was registered at clinicaltrials.gov NCT05509075.

**Results:**

Significant improvements with moderate effect size were found on memory consolidation at the short and long term only after BR-CT supplementation via the Rey Auditory Verbal Learning Test immediate (+ 20.69%) and delayed (+ 12.34%) recalls. Likewise, enhancement on both frontal lobe functions (+ 2.57%) and cognitive flexibility (+ 11.16%) were detected after BR-CT. There was no significant change (*p* < 0.05) on verbal memory of short-term digits, working memory and information processing speed. Mixed results were found on mood and anxiety through the Beck Depression Inventory-II (BDI-II) and the State-Trait Anxiety Inventory (STAI-Y1 and STAI-Y2); however, sequence and period effects were seen on STAI-Y2.

**Conclusions:**

The acute administration of a chewable BR-based supplement improves certain aspects of cognitive function in healthy females and males, particularly memory capacity and frontal skills.

## Introduction

**Fig. 1:**
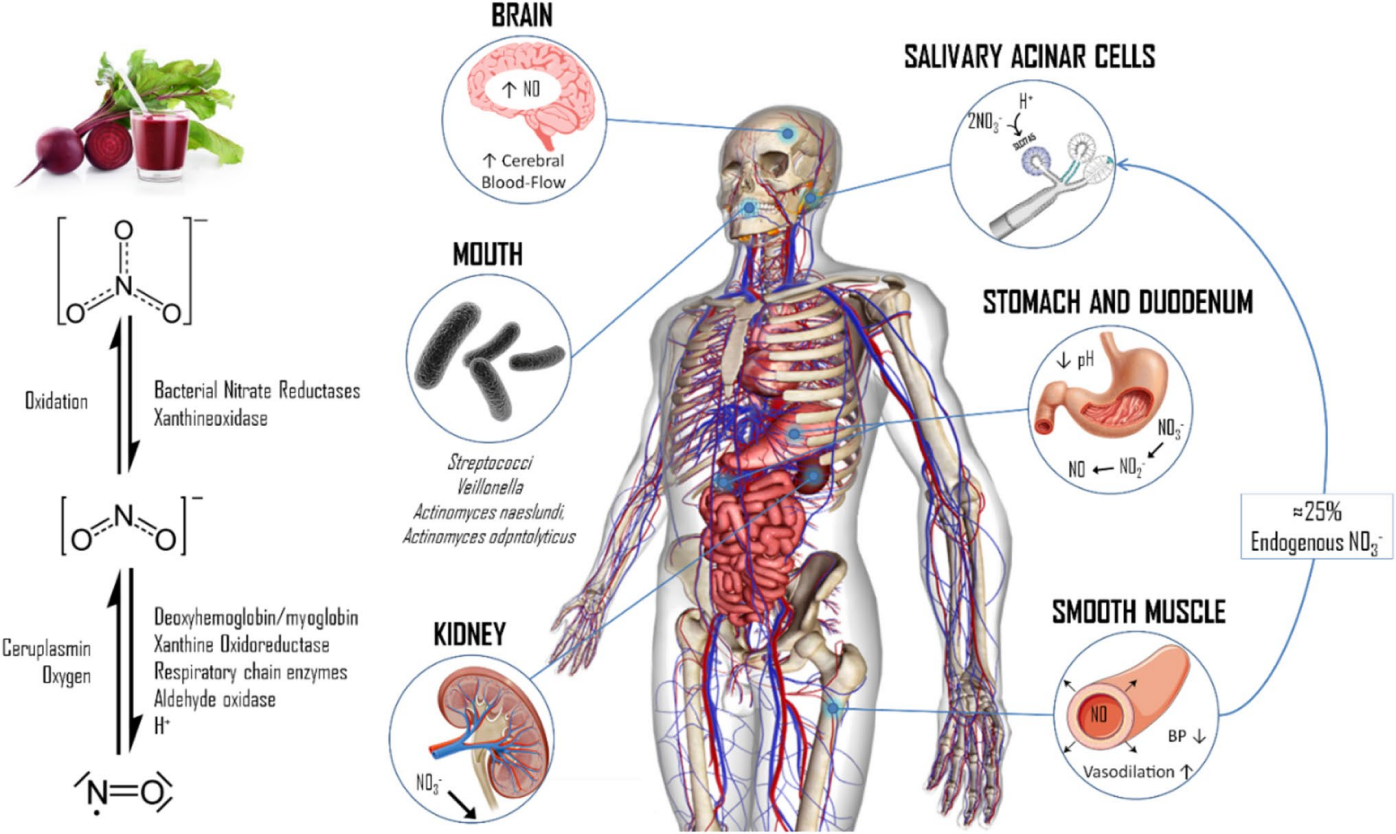
The nitrate/nitrite/nitric oxide (NO_3_^−^/NO_2_^−^/NO) pathway after dietary NO_3_^−^ ingestion. Next to BR ingestion, oral microbiota on the posterior surface of the tongue is able to reduce NO_3_^−^ to NO_2_^−^ by means of their enzymatic machinery. The strict anaerobes *Veillonella atypical* and *Veillonella dispar* are the most important NO_3_^−^ reducers; however, *Actinomyces*, *Rothia*, *Prevotella*, *Neisseria*, and *Haermophilus* are also present in the oral cavity. Even though this nonenzymatic reduction process continues in the stomach, where more NO_2_^−^ and NO are produced due to the acid environment, a considerable amount of NO_3_^−^ from blood (≈ 25%) is taken up by an electrogenic 2NO_3_^−^/H^+^ symporter called SLC17A5 (also known as sialin) in the salivary gland acinar cells [78]. Both dietary and saliva NO_3_^−^, and its reduced forms NO_2_^−^ and NO, enter directly to systemic circulation after the absorption process in the stomach and intestine. Thus, the increase of NO_3_^−^ and NO_2_^−^ concentrations in blood allow the generation of NO by either enzymatic or non-enzymatic mechanisms (such as xanthine oxidoreductase, respiratory chain enzymes, aldehyde oxidase, methemoglobin formation, protons, etc.), especially under physiologic hypoxia and low pH [79]. Because of its short half-life (1–2 ms), once NO is produced in blood, it is broken down by hemoglobin or it can diffuse into the vascular smooth muscle cells or neurons and bind to guanylyl cyclase, which allows the allosteric activation of this last and subsequent cGMP production [80]. Here, cGMP acts as a second messenger and activates PKG, which in turn can modulate smooth muscle relaxation by several interlinked mechanisms: (i) activation of K^+^ channels leading to hyperpolarization; (ii) reduction of intracellular Ca^2+^ concentration; and (iii) activation of the myosin light-chain phosphatase [81]. Finally, NO_3_^−^ is normally excreted in the urine by the kidneys. *BP* blood pressure, *NO* nitric oxide. Modified with permission from Bonilla et al. [4]

Consumption of naturally nitrate- (NO_3_^−^) rich foods such as beetroot (BR), spinach, arugula or amaranth has shown a positive effect on health and disease [1]. The increased levels of blood NO_3_^−^ after NO_3_^−^ rich foods or supplements result in the augmentation of nitrite (NO_2_^−^) concentration and higher production of nitric oxide (NO) [2]. The synthesis of NO from NO_3_^−^ is an alternative pathway to the canonical one that involves nitric oxide synthase (eNOS) using l-arginine as the main substrate [3]. NO_3_^−^ from the diet have a first conversion into NO_2_^−^ by the salivary microbiome which is then exposed to the low pH environment of the gastric acid and is reduced to NO. The significant increase in NO occurs through a process orchestrated by several tissues of the gastrointestinal tract, the blood, and the endothelium [4] (Fig. 1). It should be noted that the greatest conversion occurs in hypoxic conditions (as eNOS needs oxygen to be active) [3].

Importantly, dietary NO_3_^−^ has shown to increase cerebral blood flow in humans [5]; therefore, NO could not only reach skeletal muscle, but also cross the blood–brain barrier and act on the central nervous system [6, 7]. Indeed, the main areas in which NO_3_^−^ supplementation has been studied include those that obtain an ergogenic effect [8–11] or as regulators of blood pressure and cardiovascular health [4, 12, 13]. It is plausible that the non-enzymatic-dependent production of NO is responsible for most of the health and exercise performance benefits, although the presence of other secondary metabolites (e.g., betalains, oxalic acid, hydroxycinnamic acids) could also mediate the physiological response to NO_3_^−^ rich foods [12]. It is necessary to point out that some effects of NO_3_^−^ supplementation are a scientific controversy possibly due to the lack of standardization in the concentration (i.e., the amount of NO_3_^−^ present in the extract), the dietary source, the timing of intake [14], the salivary oral microbiome [15, 16], and individual responses based on adaptations to stress conditions (e.g., physical exercise). Moreover, there are concerns related to potential toxicity, but expert consensus suggests that dietary NO_3_^−^ supplementation up to ~ 16 mmol per day does not increase risk of cancer, methemoglobinemia hypotension, or renal injury [9]. Nowadays, it is clear that skeletal muscle concentration of NO_3_^−^ and an optimal production of NO are both critical for healthy aging and disease management [17–19].

Several neurological diseases have been associated with the age-dependent decrease of cerebral blood flow [20, 21]. This has raised the potential of NO_3_^−^ supplementation to slow the cognitive decline in elderly populations. Although BR supplementation has shown positive effects on perfusion to the brain [22, 23], the effects on cognitive performance continue being studied through the last years but remain unclear [24]. It has been reported that dietary NO_3_^−^ might potentially improve cognitive performance in healthy adults [5] and in type-2 diabetes patients [25]. The systematic review performed by Stanaway et al. not only showed a lack of studies measuring cognitive performance-related variables (only 3 of 12 included studies), but also reported mixed findings [26]. Consistent with previous findings, a recent randomized, double-blind crossover trial showed only certain improvements of cognitive performance in the Stroop test, but not in choice reaction test or rapid visual information processing after BR supplementation [27]. The authors suggested that the positive effects of BR might be only present when a large degree of cognitive difficulty is imposed. Notwithstanding, it has been shown recently that acute dietary NO_3_^−^ supplementation via red spinach extract or BR juice has no important effects on cognitive performance, in resistance-trained males or in healthy participants exercising at moderate or very high simulated altitudes, respectively. Currently, there is no consensus about the effects of dietary NO_3_^−^ or BR supplementation on cognitive function in the elderly population [28–30] or in athletes [9]. Thus, the aim of this study was to assess the acute effects of a chewable BR-based supplement on cognitive performance (memory and executive function) using a neuropsychological battery of tests in apparently healthy female and male individuals.

## Methods

### Trial design

**Fig. 2:**
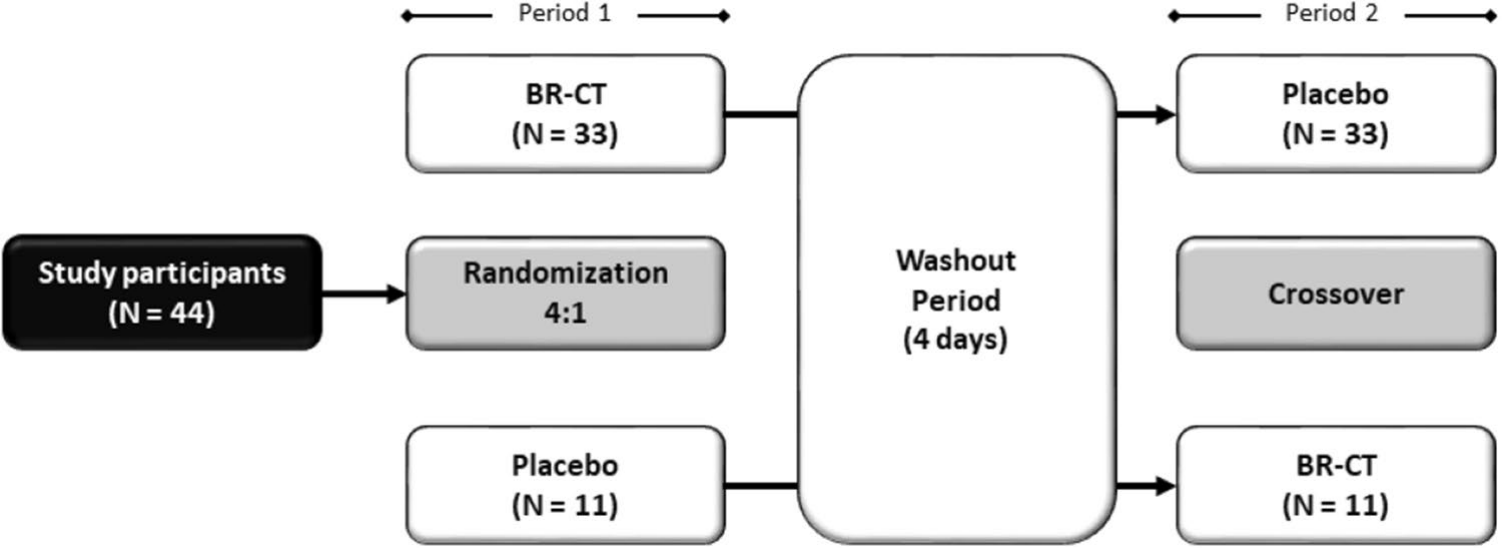
Schematic representation of the study design. *BR-CT* beetroot chewing tablets

This study was a double-blind randomized placebo-controlled two-period crossover clinical trial to assess cognitive performance after the administration of a chewable BR-based supplement. The neuropsychological testing battery was applied at the end of each period. The experimental procedures were conducted following those established in the extension of the Consolidated Standards of Reporting Trials (CONSORT) for randomized crossover trials [31]. A 4-day washout period was used to reduce the carryover effect [32]. The study design is schematized in Fig. 2.

### Participants

A total of 70 participants volunteered to participate in this clinical trial. Subjects were suitable for eligibility if they were: (i) apparently healthy; (ii) 18–60 years of age; (iii) non-smokers; (iv) without overt pathologies; (v) not taking drugs regularly; and (vi) committed to follow instructions of the research team at the testing time. The individuals were informed about the experimental protocol and all provided informed written consent before participating. The study protocol was approved by the Regional Ethical Committee (Center Italy Section) N12017052018.

### Settings and location

The administration of the placebo or the active tablets and the application of the neuropsychological testing battery were performed within the Department of Medical and Surgical Sciences facilities at the University of Magna Grecia, Catanzaro Italy. The battery was administered to each participant in the same order and in two single test sessions. Parallel test forms were administered to verify the learning effect. All tests were provided by the same neuropsychologist (with more of than 10 years of experience).

### Intervention

The participants that met the inclusion criteria were administered four chewable tablets with the same size, color, and shape of either active product or placebo 90 min before the neuropsychological test. The participants were asked to dissolve the tablets in the mouth (to favor the contact with the salivary microbiome) and to swallow saliva as many times as possible. The BR-based chewable tablets (BR-CT) contained 3 g of a *Beta vulgaris* extract (RedNite^®^, Enovate Biolife, Wilmington, DE) with standardized NO_3_^−^ concentration range between 1.5 and 2.75% (equals to 45–82.5 mg of NO_3_^−^). NO_3_^−^ supplementation with RedNite^®^-based products has already shown clinically significant effects on neuromuscular efficiency in healthy males [33]. The BR-CT also contains 200 mg of vitamin C and 100 mg betaine, both were used in those low doses to synergize the conversion of NO_3_^−^ into NO avoiding its conversion in nitrosamine, as well as *Citrus sinensis* (blood orange) extract as flavoring, sucralose as sweetener, and excipients and stabilizers for the constitution of the tablet.

The placebo contained maltodextrin instead of the active components and had the same shape, color, and taste. The participants were requested to avoid food, not to rinse the mouth, not to engage in strenuous physical activity in the 2-h period before taking the chewable tablets, and to avoid stimulating substances (i.e., caffeine or theophylline) 6 h prior to the intervention. The formulations were produced by Laboratori Plants Group (Pace del Mela ME, Italy).

### Outcomes

#### Neuropsychological assessment

The battery was administered to each participant in the same order and in two single test sessions and parallel forms of tests were administered to check the learning effect in a testing session lasting from 30 to 40 min. The psychologist was not informed about the treatment, the BR-CT and placebo were of the same color, and neither the subject nor the researcher knew which was the placebo and which was the BR-CT The following validated tests were administered 90 min after taking BR-CT or placebo:

- The Rey Auditory Verbal Learning Test (RAVLT) which includes the assessment of immediate (RAVLT_I) and delayed recalls (RAVLT_D) to evaluate short-term and long-term verbal memory, respectively [34–36]. In this study, parallel forms of RAVLT's words were used (e.g., tenda, tamburo, etc., and violino, bastone, etc.).
- The Digit Span Forward (Digit Span F) and Backward (Digit Span B) tasks to evaluate the memory span: the verbal memory of short-term digits and working memory standardized for Italian population [37, 38] (these are subsets of the Wechsler Adult Intelligence Scale-Fourth Edition [WAIS-IV] [39]).
- The Symbol Digit Modality Test (SDMT) to measure attention and processing speed [40]. In this study, the parallel forms were used [41].
- The Frontal Assessment Battery (FAB) for a fast screening of the main frontal skills such as abstract reasoning, mental flexibility, motor programming, executive control of action, and inhibitory control [42, 43].
- The Controlled Oral Word Association Test (COWAT/FAS), used as a measure of lexical assets, tests the ability to access the lexicon and cognitive flexibility [44, 45]. In this study, parallel forms were used (i.e., letters F, A, S and E, G, L).
- The self-assessment questionnaires to investigate mood included the 2nd edition of the Beck Depression Inventory (BDI-II) [46, 47] used to assess the depressive symptoms of the participants and the State–Trait Anxiety Inventory-Form Y1 and 2 (STAI-Y1; STAI-Y2) with 40 entries [48, 49]. The STAI provides scores for two scales (20 and 20) distinguishing between a person's status and trait anxiety levels. It should be noted that parallel forms were not used for mood questionnaires.
- The Retrospective Prospective Memory Questionnaire (PRMQ) which is a self-report questionnaire that provides information for both the prospective (PRMQ-P) and retrospective (PRMQ-R) scales [50, 51]. These non-parallel tests were applied to investigate the perception of subjects in any modifications of cognitive functions [52].

### Sample size

The sample size was calculated, based on the percentage change of the primary outcomes. Previous literature in adults has shown significant differences in cognitive performance tests between 5 and 12% when comparing BR supplementation versus placebo [22, 25]. Therefore, we used a minimum effect of interest equal to 9% (8 SD), a type I error rate (*α*) of 0.05 and with a power of 0.8, and obtained a sample size of ten per group. Considering our randomization ratio, we needed 40 participants for the BR-CT and 10 for placebo. To allow for attrition, 70 participants were enrolled.

### Randomization and sequence generation

Unequal randomization (4:1 randomization of active to placebo) was used to give more power for pairwise comparisons and detect adverse events due to the larger sample size in the active group. In addition, unequal randomization allows more variables to be tested and better recruitment. A random allocation sequence was computer generated (https://www.randomizer.org/). We performed a two-period crossover with a treatment sequence AB:BA, which means that participants allocated to the AB study arm receive treatment A first, followed by treatment B, and vice versa in the BA arm. The AB/BA is not only usable, but also is considered the most efficient two-period two-treatment design [53]. To exclude a priori the carryover effects, we used a 4-day washout period that was set at three to four times the blood plasma elimination half-life for NO_3_^−^ (5–8 h) [54, 55].

### Blinding

This was a double-blinded clinical trial because participants and those assessing the outcomes were blinded to intervention. The placebo tablets were identical in size, shape, color and taste, but contained inactive ingredients (maltodextrin). Every person involved in the study received two cruets, marked with a code provided by the manufacturer and unknown to us and to the subjects, inside three tablets each. Both, the cruets and the tablets, were identical to make distinguishing between them impossible. Therefore, participants itself choose was ensured. The placebo formulation was manufactured by the Laboratori Plants Group (Pace del Mela ME, Italy).

### Statistical methods

The descriptive statistics are expressed as mean and standard deviation (SD) unless otherwise indicated. Following the extension of CONSORT guidelines for randomized crossover trials [31], we based our analysis on paired data (within-participant comparison) using an intention-to-treat approach. As it has been recommended for crossover trials [56], and before estimating the treatment effects, the results were analyzed for each intervention in each period [57] and the sequence and period effects were estimated. It should be noted that the carryover effect was avoided by using a washout period of sufficient duration (4 days) [58].

Based on current recommendations to improve data analysis practices, we implemented an estimation approach following analytical procedures reported in previous articles published by the DBSS Research Divison [59, 60]. Estimation statistics helps to obtain more thoughtful interpretation and more balanced evaluation of evidence [61]. To determine statistical significance in the analysis of paired data, we examined the 95% confidence intervals (CIs) for the difference (Δ) between the placebo and BR-CT. If the 95% CI excludes zero, the difference will attain significance at the *p* < 0.05 level. Effect size was calculated as unbiased Cohen’s *d* (d_unb_), considering a result of ≤ 0.2 as a small, 0.5 as a moderate, ≥ 0.8 as a large effect, and ≥ 1.30 as a very large effect [62]. Estimation plots were generated to display the paired data at placebo and after BR-CT supplementation. Percentages of change were calculated according to the formula: (BR-CT − placebo)/placebo) × 100. To help with the planning and commissioning of future crossover trials, we also report the correlation coefficient for each primary outcome during the within-participant comparison. The same statistical procedure was performed in the paired-data analysis for each sequence. The sequence effect was estimated by comparing the means of the dependent variables in the AB and BA sequences.

In the analysis for each intervention in each period, we used the Yuen–Dixon test [63] with 20% trimmed means (*μ*_t_) and 20% winsorized standard deviations (*σ*_w_) as a robust statistical method for unequal-sized samples (e.g., BR-CT [*n* = 33] versus placebo [*n* = 11]). This robust statistics not only can be applied to factorial-type experimental designs, but also provide broader control of Type I error when variances are not equal [64]. A difference-in-differences (DID) analysis was performed to estimate the period effect by comparing changes in the outcome variables between each period. Statistical analyses were performed using the Exploratory Software for Confidence Intervals [65].

## Results

### Participant flow

**Fig. 3:**
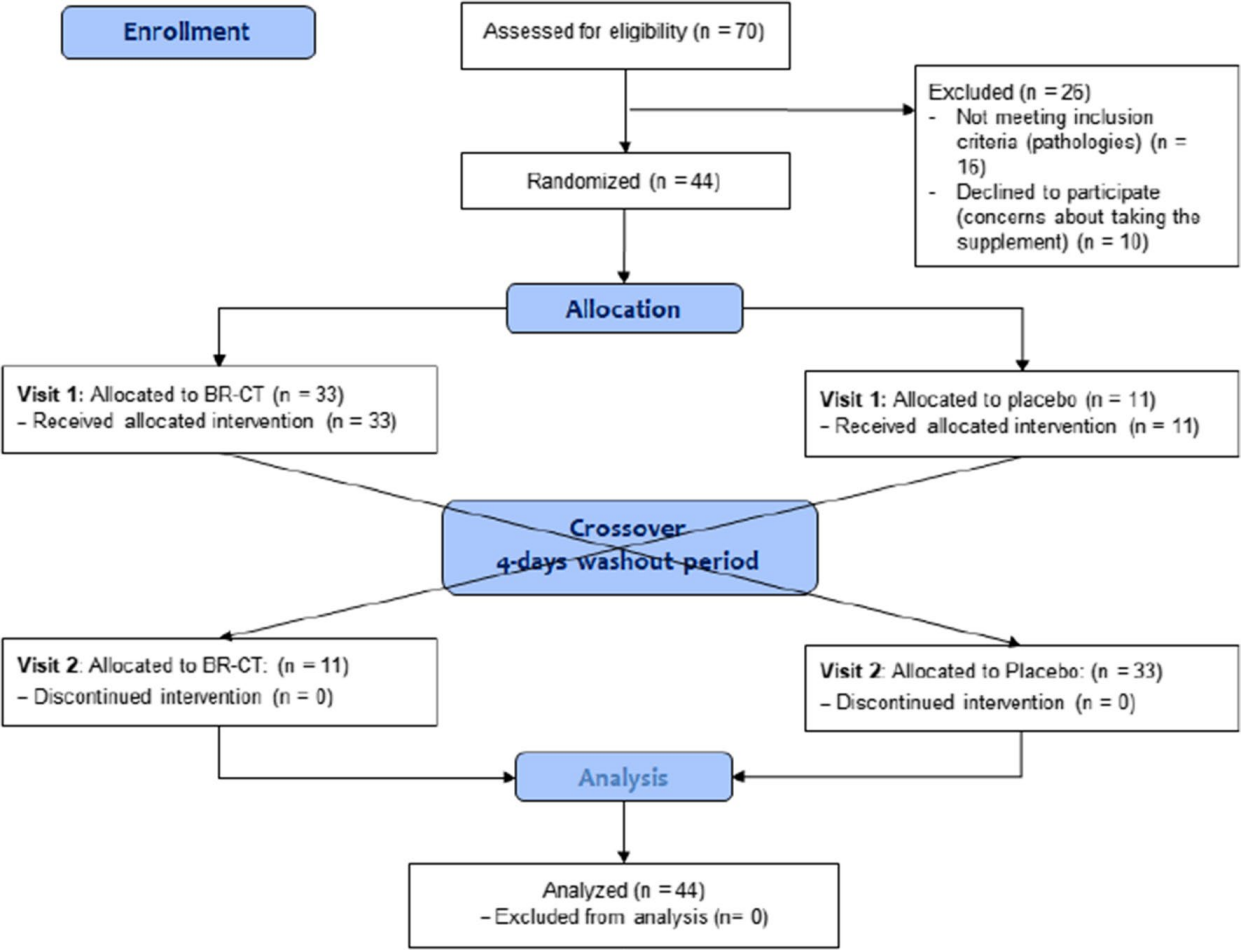
CONSORT flow diagram for crossover trials

After the call to participate, 70 participants were potentially eligible. However, 26 individuals were excluded from this study due to the presence of pathologies (*n* = 16), while others had concerns about taking the supplement and declined to participate (*n* = 10). Hence, 44 nonsmoking healthy participants (18–60 years of age) without overt pathologies completed this clinical trial. Figure 3 shows the CONSORT flow diagram modified for randomized crossover trial designs.

### Baseline data

Table 1 resumes the characteristics of participants. The supplementation with BR-CT was well tolerated among all participants and there were no reported adverse effects, acute or 1 month after the study.

**Table 1:** Characteristics of the participants

	All (*n* = 44)	BR-CT first (*n* = 33)	Placebo first (*n* = 11)
Age (years)	32.7 (12.5)	29.6 (8.1)	33.7 (13.6)
Women	24 (54.55%)	14 (42.42%)	10 (90.90%)
Men	20 (45.45%)	19 (57.57%)	1 (9.09%)
Body mass (kg)	66.3 (9.0)	60.4 (8.0)	68.3 (8.5)
Stature (cm)	170 (9.2)	163.2 (6.1)	172 (9.0)
BMI (kg/m^2^)	22.8 (1.4)	22.6 (1.8)	22.9 (1.2)
Education			
High school	24 (54.55%)	16 (48.48%)	8 (72.73%)
College	17 (38.64%)	14 (42.42%)	3 (27.27%)
Post-graduate	3 (6.82%)	3 (9.09%)	0 (0.00%)
Recreational sports practice			
Yes	18 (40.91%)	15 (45.45%)	3 (27.27%)
No	26 (59.09%)	18 (54.55%)	8 (72.73%)
Coffee consumption			
Yes	34 (77.37%)	27 (81.82%)	7 (63.64%)
No	10 (22.73%)	6 (18.18%)	4 (36.36%)

Data are expressed as mean (standard deviation) unless otherwise indicated (percentage)

### Outcomes and estimation

The results of the within-participant comparison (analysis on paired data) are expressed as Δ (SD) [95% CI]; d_unb_ [95% CI] and presented in Table 2. The results on the RAVLT_D and RAVLT_I tests after BR-CT consumption showed higher statistically significant values with moderate effect size in comparison to placebo (12.34% and 20.69%, respectively). There were no significant differences between BR-CT and placebo, neither on Digit Span both forward (0.02 (1.19) [− 0.33, 0.38]; 0.021 [− 0.31, 0.35]) and backward (0.06 (1.10) [− 0.26, 0.40]; 0.069 [− 0.26, 0.40]), nor on the SDMT (− 4.43 (15.79) [− 9.23, 0.37]; 0.320 [− 0.67, 0.02]) tests. The FAB and COWAT/FAS tests were significantly improved with small effect size after BR-CT supplementation (2.57% and 11.16%, respectively). Although the results of the BDI test revealed significantly higher rates of depression after BR-CT compared to placebo (1.54 (3.63) [0.44, 2.64]; 0.261 [0.07, 0.45]), the STAI-Y1 and STAI-Y2 did not show significant differences on the anxiety levels between conditions. Finally, there were no differences in memory performance via the prospective, retrospective, and total PMRQ test (*p* > 0.05).

**Table 2:** Paired-data analysis for BR-CT versus placebo

Variable	Placebo (*n* = 44) $\overline{X}$ (SD)	BR-CT (*n* = 44) $\overline{X}$ (SD)	Δ (SD) [95% CI]	*d*_unb_ *δ* [95% CI]	*p* value	Correlation
RAVLT_D	42.68 (7.68)	47.95 (8.67)	5.27 (6.52) [3.28, 7.25]	0.632 [0.36, 0.91]	< 0.001	0.69
RAVLT_I	8.36 (2.85)	10.09 (2.59)	1.72 (3.28) [0.72, 2.72]	0.622 [0.25, 1.01]	0.001	0.27
Digit span F	5.56 (1.12)	5.59 (0.97)	0.02 (1.19) [– 0.33, 0.38]	0.021 [– 0.31, 0.35]	0.900	0.37
Digit span B	3.65 (1.01)	3.72 (0.92)	0.06 (1.10) [– 0.26, 0.40]	0.069 [– 0.26, 0.40]	0.685	0.35
SDMT	69.0 (12.01)	64.56 (15.02)	–4.43 (15.79) [– 9.23, 0.37]	0.320 [– 0.67, 0.02]	0.070	0.33
FAB	15.90 (1.42)	16.31 (1.11)	0.40 (1.2) [0.02, 0.79]	0.314 [0.01, 0.61]	0.037	0.53
COWAT/FAS	32.52 (9.67)	36.15 (8.00)	3.63 (6.79) [1.57, 5.70]	0.403 [0.16, 0.65]	0.001	0.72
BDI	5.79 (5.39)	7.34 (6.19)	1.54 (3.63) [0.44, 2.64]	0.261 [0.07, 0.45]	0.007	0.81
STAI Y1	33.95 (7.82)	35.43 (10.52)	1.47 (6.71) [– 0.56, 3.51]	0.157 [– 0.05, 0.37]	0.152	0.77
STAI Y2	38.59 (9.53)	39.34 (10.32)	0.75 (5.15) [– 0.81, 2.31]	0.074 [– 0.07, 0.22]	0.340	0.87
PMRQ P	19.31 (5.27)	19.68 (5.93)	0.36 (3.48) [– 0.69, 1.42]	0.064 [– 0.12, 0.24]	0.493	0.81
PMRQ R	18.43 (4.52)	19.09 (4.92)	0.65 (3.01) [– 0.25, 1.57]	0.137 [– 0.05, 0.32]	0.154	0.80
PMRQ TOT	37.75 (9.31)	39.0 (10.15)	1.25 (6.11) [– 0.60, 3.11]	0.126 [– 0.06, 0.31]	0.183	0.81

Data is presented as mean ($\overline{X}$) and standard deviation (SD). The difference will attain significance at the *p* < 0.05 level if the 95% CI excludes zero

*CI* confidence interval, *d*_unb_ (*δ*) unbiased Cohen’s *d*

**Fig. 4:**
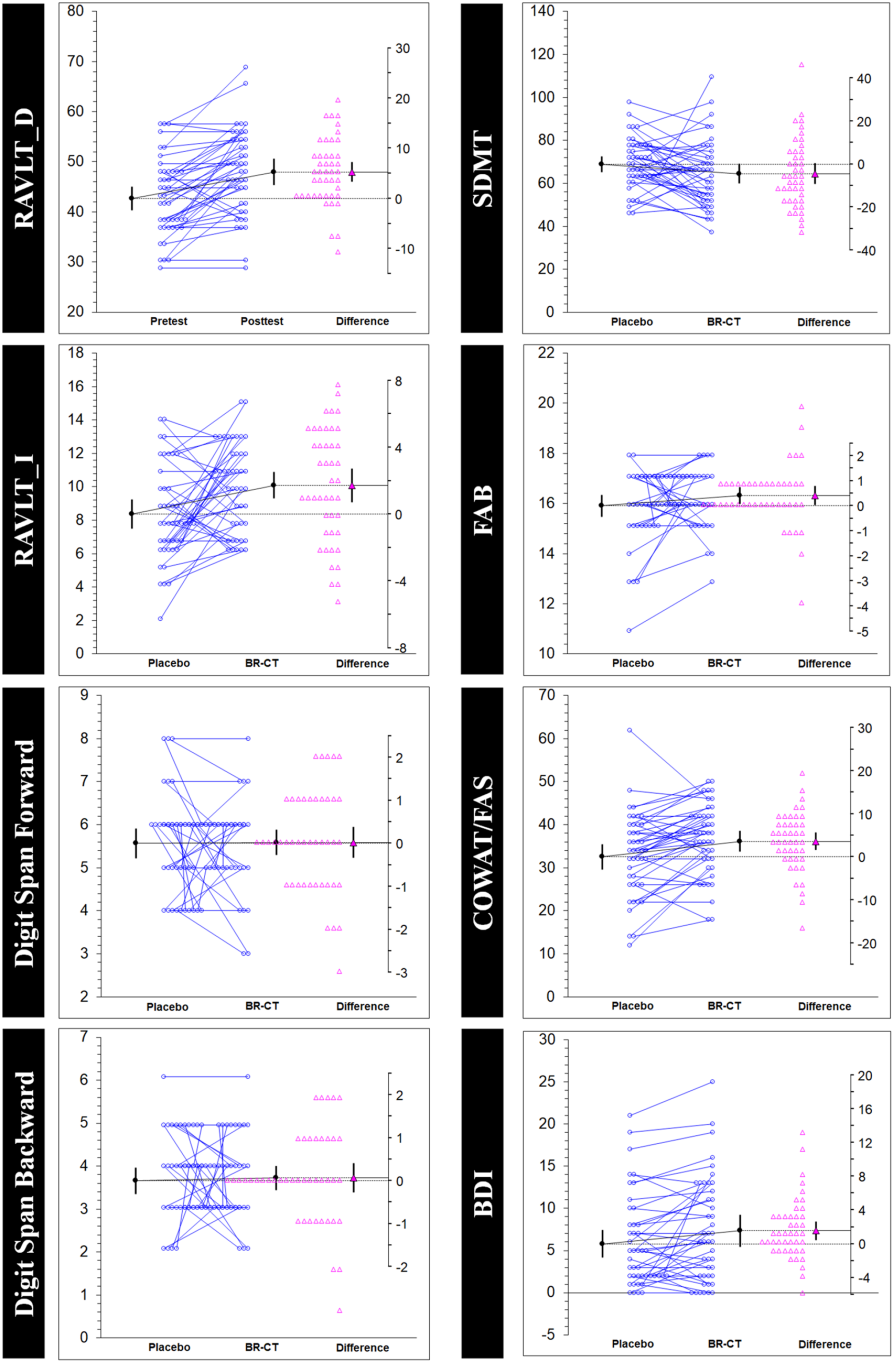

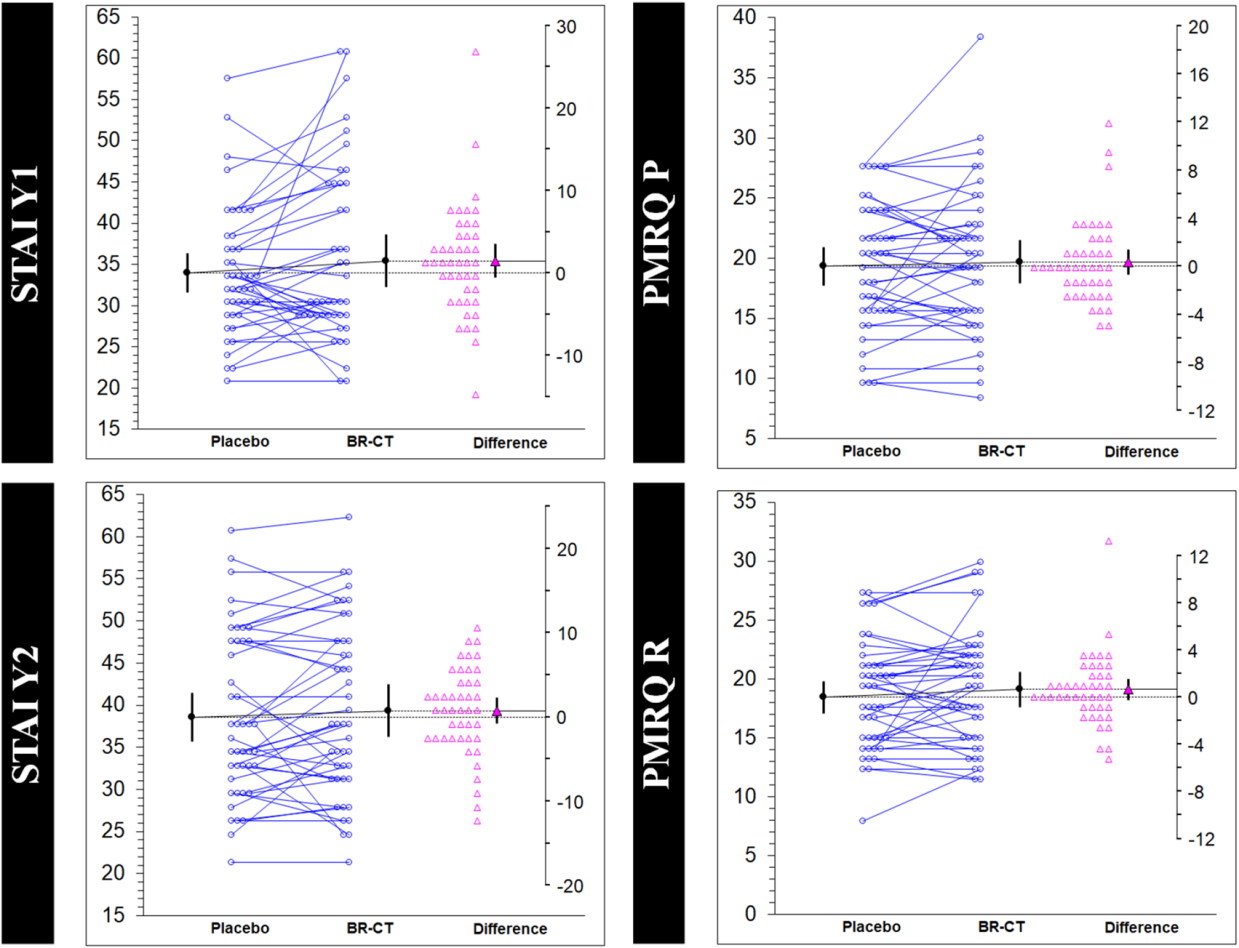
Estimation plots examining the within-participant comparisons on the study variables. Paired data from placebo (maltodextrin) and experimental (BR-CT) conditions are shown as small circles joined by blue lines. The differences between the placebo and treatment means are plotted on a floating difference axis whose zero is aligned with the placebo mean. The filled pink triangle marks the difference on that axis and the 95% CI on that difference is displayed. The differences are shown as open triangles on the difference axis

Figure 4 shows the estimation plots with raw data of each paired set of observations connected by a line to compare the placebo and BR-CT conditions. To complement the analysis, the changes for each sequence with the corresponding sequence effects are reported in Table 3. A higher number of variables showed significant differences in sequence AB. While BR-CT supplementation revealed significant improvements with large effect size on the RAVLT_D (– 6.12 (11.52) [– 10.2, – 2.0]; 0.740 [1.27, 0.23]), RAVLT_I (– 2.51 (4.07) [– 3.95, – 1.07]; 0.926 [1.51, 0.36]) and COWAT/FAS (– 6.27 (11.79) [– 10.4, – 2.09]; 0.733 [1.26, 0.23]) tests, the consumption of placebo showed better outcomes on SDMT (10.30 (18.51) [3.73, 16.86]; 0.775 [0.26, 1.31]) and less anxiety levels via BDI (– 2.36 (6.54) [– 4.68, – 0.04]; 0.473 [0.95, 0.008]). On the other hand, only two variables showed significant difference in sequence BA, both favoring the BR-CT condition: Digit Span B (0.90 (1.04) [0.20, 1.61]; 0.752 [0.14, 1.45]) and SDMT (13.18 (14.00) [3.77, 22.59]; 1.156 [0.25, 2.01]). There was a sequence effect by randomization in the AB/BA sequence only on STAI Y2 (*p* = 0.011). No other sequence effects were found.

**Table 3:** Paired-data analysis for each sequence

Variable	Sequence AB				Sequence BA				Sequence effect
	BR-CT (*n* = 33) $\overline{X}$ (SD)	Placebo (*n* = 33) $\overline{X}$ (SD)	Δ (SD) [95% CI]	*d*_unb_ *δ* [95% CI]	Placebo (*n* = 11) $\overline{X}$ (SD)	BR-CT (*n* = 11) $\overline{X}$ (SD)	Δ (SD) [95% CI]	*d*_unb_ *δ* [95% CI]	*p* value
RAVLT_D	47.97 (8.82)	41.84 (7.24)	– 6.12 (11.52) [– 10.2, – 2.0]*	0.740 [1.27, 0.23]	45.18 (8.76)	47.90 (8.60)	2.72 (6.66) [– 1.75, 7.20]	0.290 [0.16, 0.78]	0.421
RAVLT_I	10.21 (2.58)	7.69 (2.72)	– 2.51 (4.07) [– 3.95, – 1.07]*	0.926 [1.51, 0.36]	10.36 (2.33)	9.72 (2.72)	– 0.63 (3.10) [– 2.72, 1.45]	0.231 [–0.97, 0.48]	0.095
Digit Span F	5.45 (1.03)	5.72 (0.97)	0.27 (1.37) [– 0.21, 0.76]	0.265 [– 0.20, 0.74]	5.09 (1.44)	6.0 (0.63)	0.90 (1.04) [0.20, 1.61]*	0.752 [0.14, 1.45]	0.859
Digit Span B	3.66 (0.95)	3.75 (1.06)	0.09 (1.42) [– 0.41, 0.59]	0.088 [– 0.39, 0.57]	3.36 (0.80)	3.90 (0.83)	0.54 (1.03) [– 0.15, 1.24]	0.614 [– 0.14, 1.45]	0.752
SDMT	60.66 (13.45)	70.96 (12.49)	10.30 (18.51) [3.73, 16.86]*	0.775 [0.26, 1.31]	63.09 (8.34)	76.27 (13.79)	13.18 (14.00) [3.77, 22.59]*	1.156 [0.25, 2.01]	0.217
FAB	16.36 (1.02)	16.0 (1.29)	–0.36 (1.38) [– 0.85, 0.12]	0.303 [– 0.72, 0.10]	15.63 (1.80)	16.18 (1.40)	0.54 (0.68) [0.08, 1.00]	0.312 [0.04, 0.62]	0.393
COWAT/FAS	37.48 (7.57)	31.21 (9.06)	– 6.27 (11.79) [– 10.4, – 2.09]*	0.733 [1.26, 0.23]	36.45 (10.7)	32.18 (8.26)	– 4.27 (5.47) [– 7.95, – 0.59]	0.410 [0.82, 0.04]	0.989
BDI	7.30 (5.31)	4.93 (4.39)	– 2.36 (6.54) [– 4.68, – 0.04]*	0.473 [0.95, 0.008]	8.36 (7.32)	7.45 (8.62)	– 0.90 (2.02) [–2.26, 0.44]	0.105 [0.26, 0.04]	0.212
STAI Y1	34.45 (9.46)	32.93 (7.48)	– 1.51 (10.97) [–5.40, 2.37]	0.173 [–0.61, 0.26]	37.0 (8.370)	38.36 (13.29)	1.36 (8.13) [– 4.10, 6.83]	0.113 [– 0.31, 0.55]	0.083
STAI Y2	38.51 (9.25)	36.33 (8.76)	– 2.18 (12.29) [– 6.54, 2.17]	0.236 [–0.71, 0.22]	45.36 (8.82)	41.81 (13.24)	– 3.54 (5.93) [–7.53, 0.44]	0.291 [– 0.64, 0.03]	0.011 †
PMRQ P	19.84 (6.01)	19.27 (5.39)	– 0.57 (8.32) [– 3.52, 2.37]	0.098 [–0.59, 0.39]	19.45 (5.16)	19.18 (5.92)	– 0.27 (3.03) [– 2.31, 1.76]	0.045 [– 0.37, 0.27]	0.863
PMRQ R	19.03 (5.19)	18.24 (4.86)	– 0.78 (7.41) [– 3.41, 1.84]	0.153 [– 0.66, 0.34]	19.0 (3.43)	19.27 (4.22)	0.27 (2.24) [– 1.23, 1.77]	0.065 [– 0.27, 0.41]	0.672
PMRQ TOT	39.18 (10.34)	37.51 (9.80)	– 1.66 (15.11) [– 7.02, 3.69]	0.161 [– 0.67, 0.34]	38.45 (8.02)	38.45 (10.01)	0.0 (4.47) [– 3.00, 3.00]	0.000 [– 0.29, 0.29]	0.965

Data is presented as mean ($\overline{X}$) and standard deviation (SD). The within-individual differences in each sequence are shown as Δ. The difference will attain significance at the *p* < 0.05 level if the 95% CI excludes zero. The sequence effect was calculated by comparing the means of the dependent variables in the AB and BA sequences

*CI* confidence interval, *d*_*unb*_* (δ)* unbiased Cohen’s *d*, *RAVLT* Rey Auditory Verbal Learning Test, *FAB* Frontal Assessment Battery, *FAB* Frontal Assessment Battery, *VFT* Verbal Fluency Test, *F* forward, *BW* backward

*Statistically significant change (*p* < 0.05)

^†^Statistically significance difference (*p* < 0.05 of the two-tailed *p* value)

**Fig. 5:**
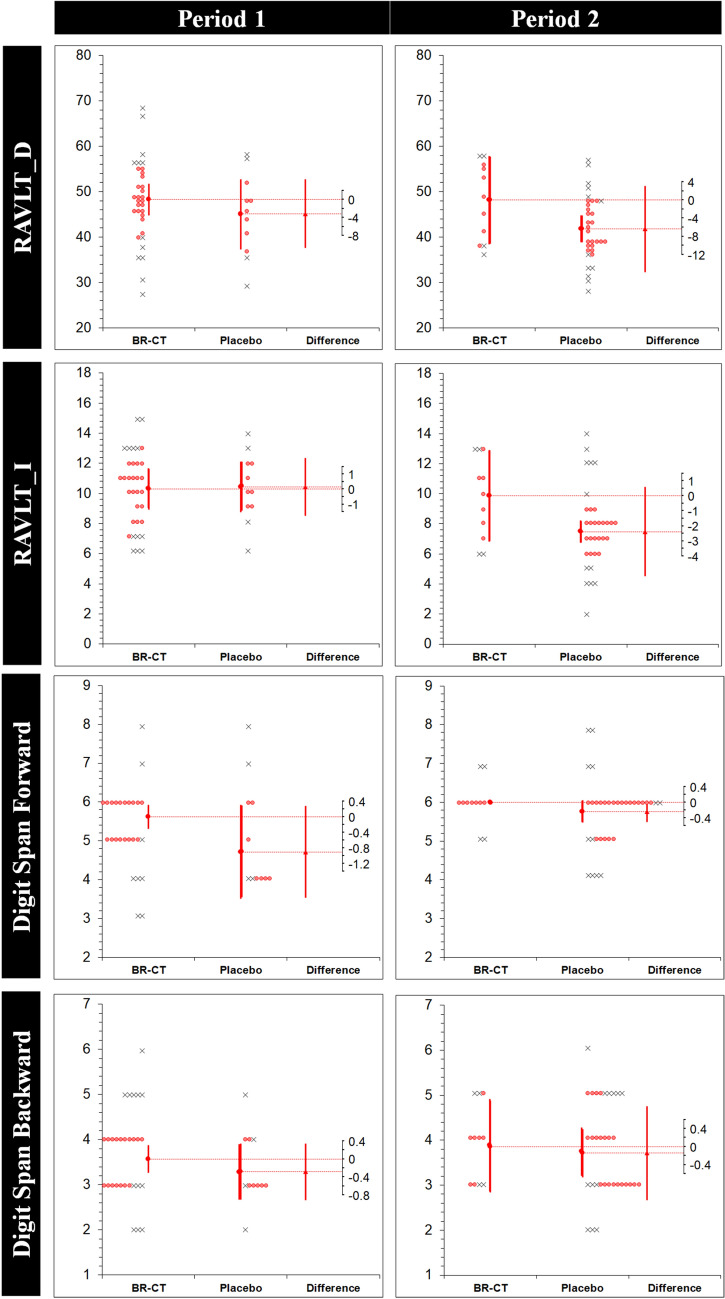

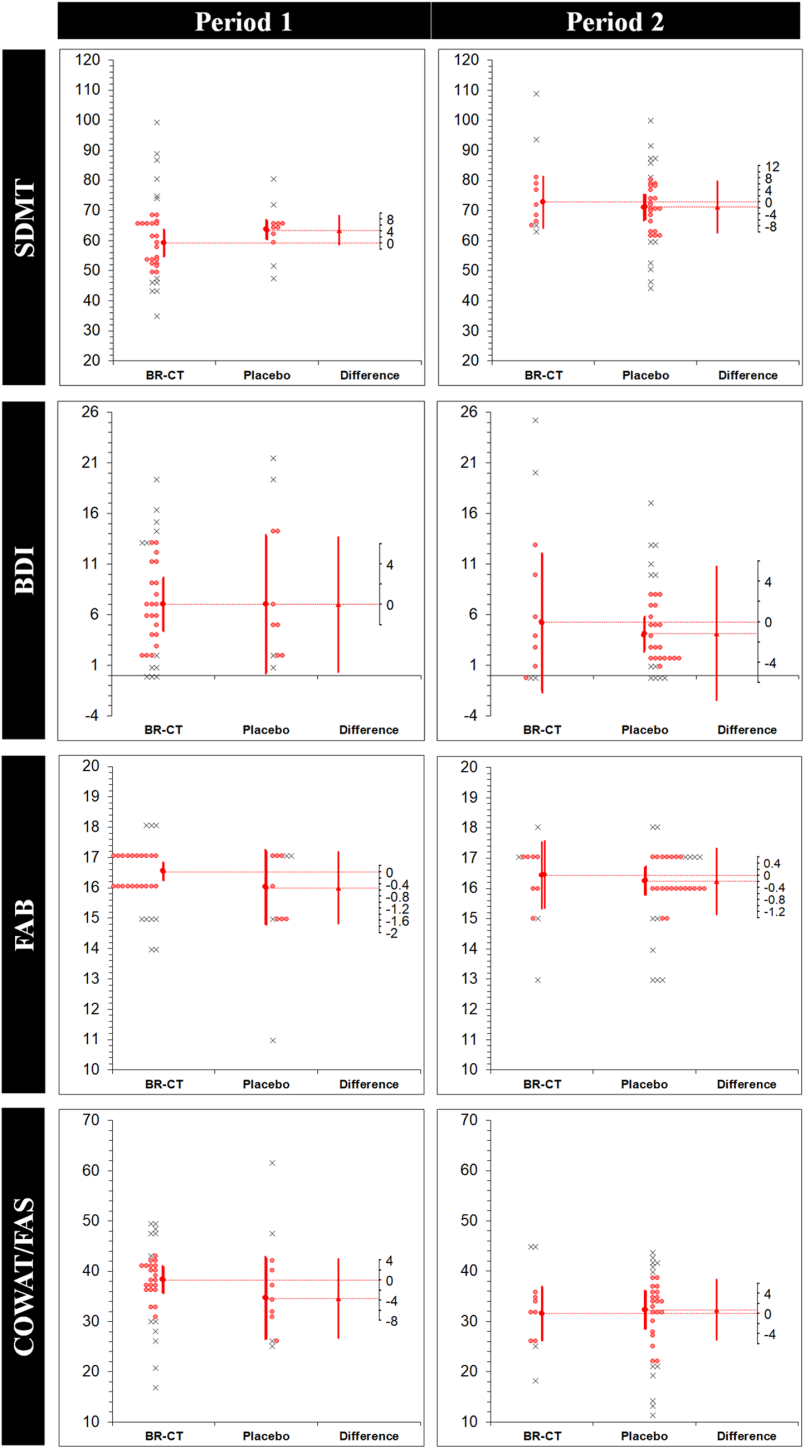

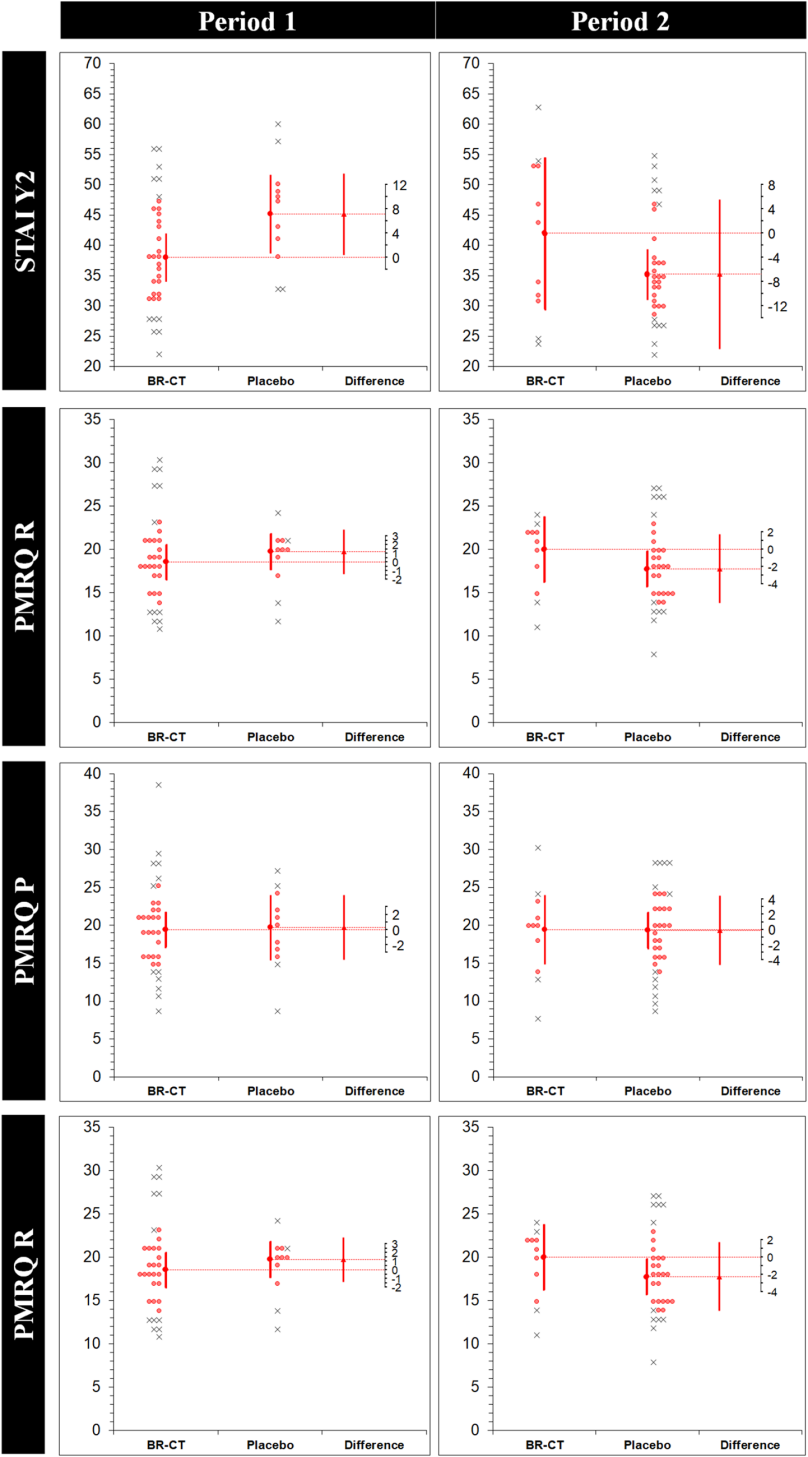

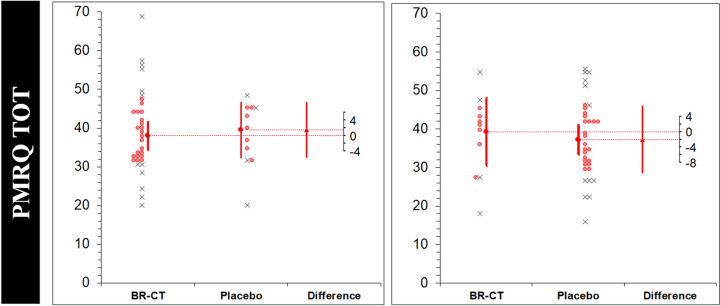
Estimation plots examining the effect of BR-CT on the study variables. Individual participants from each group are shown. Removed data points are displayed as crosses, while retained points are red. The large circles with error bars represent each group trimmed mean with their 95% confidence intervals. The difference between the experimental (BR-CT) and placebo (maltodextrin) trimmed means is plotted on a floating difference axis. The filled red triangle marks the difference between groups on that axis and the 95% CI on that difference is displayed. *BR-CT* beetroot chewing tablets

The results of the robust analysis (ES_t_ (MoE_Δ_) [95% CI]) for each intervention in each period with the corresponding period effects (DID [95% CI]; *p* value) are shown in Table 4. Significantly higher anxiety levels were found on the placebo condition through STAI Y2 (7.14 (6.74) [0.39, 13.8]); however, a period effect was found for this variable (– 12.3 [– 21.7, – 2.9]; 0.011). Figure 5 shows the estimation plots examining the effect of BR-CT on the study variables in each period. Finally, there was a direct effect of the treatment (BR-CT) only on long-term memory performance via RAVLT_D (*p* = 0.032).

**Table 4:** Robust analysis for each intervention in each period

Variable	Period 1			Period 2			Period effect	Treatment effect
	BR-CT (*n* = 33) *μ*_t_ (*σ*_w_)	Placebo (*n* = 11) *μ*_t_ (*σ*_w_)	ES_t_ (MoE_Δ_) [95% CI]	BR-CT (*n* = 11) *μ*_t_ (*σ*_w_)	Placebo (*n* = 33) *μ*_t_ (*σ*_w_)	ES_t_ (MoE_Δ_) [95% CI]	DID [95% CI] *p* value	*p* value
RAVLT_D	48.28 (5.62)	45.14 (6.12)	– 3.14 (7.56) [– 10.7, 4.42]	48.14 (7.90)	41.76 (4.88)	– 6.38 (9.53) [– 15.9, 3.15]	– 3.27 [– 11.3, 4.77] 0.421	0.032 ‡
RAVLT_I	10.28 (2.24)	10.42 (1.36)	0.14 (1.92) [– 1.77, 2.06]	9.85 (2.46)	7.47 (1.20)	– 2.38 (2.96) [– 5.34, 0.57]	– 2.18 [– 4.75, 0.38] 0.095	0.150
Digit Span F	5.61 (0.50)	4.71 (0.98)	– 0.90 (1.17) [– 2.08, 0.27]	6.0 (0.00)	5.76 (0.47)	– 0.23 (0.27) [– 0.51, 0.03]	0.09 [– 0.92, 1.10] 0.859	0.214
Digit Span B	3.57 (0.50)	3.28 (0.50)	– 0.28 (0.62) [– 0.91, 0.34]	3.85 (0.83)	3.71 (0.88)	– 0.14 (1.04) [– 1.18, 0.90]	0.15 [– 0.79, 1.10] 0.752	0.343
SDMT	59.09 (7.38)	63.42 (2.66)	4.33 (4.97) [– 0.64, 9.30]	72.85 (6.99)	71.19 (7.35)	– 1.66 (8.78) [– 10.4, 7.11]	– 7.72 [– 20.0, 4.63] 0.217	0.645
FAB	16.52 (0.50)	16.0 (1.00)	– 0.52 (1.2) [– 1.72, 0.67]	16.42 (0.90)	16.23 (0.79)	– 0.19 (1.11) [– 1.30, 0.92]	0.54 [– 0.71, 1.80] 0.393	0.156
COWAT/FAS	38.19 (4.53)	34.57 (6.63)	– 3.61 (8.03) [– 11.6, 4.41]	31.57 (4.47)	32.33 (6.60)	0.76 (6.06) [– 5.30, 6.82]	0.06 [– 8.43, 8.55] 0.989	0.641
BDI	7.0 (4.39)	7.0 (5.50)	0.00 (6.71) [– 6.71, 6.71]	5.28 (5.55)	4.14 (2.85)	– 1.14 (6.66) [– 7.80, 5.52]	– 3.57 [– 9.23, 2.08] 0.212	0.610
STAI Y1	32.42 (6.35)	37.57 (5.77)	5.14 (7.30) [– 2.15, 12.4]	38.42 (11.4)	32.14 (3.28)	– 6.28 (13.6) [– 19.9, 7.40]	– 7.97 [– 16.9, 1.04] 0.083	0.527
STAI Y2	38.0 (6.54)	45.14 (5.19)	7.14 (6.74) [0.39, 13.8]*	42.0 (10.1)	35.23 (6.84)	– 6.76 (12.3) [– 19.0, 5.57]	– 12.3 [– 21.7, – 2.9] 0.011^†^	0.774
PMRQ P	19.42 (3.84)	19.71 (3.37)	0.28 (4.28) [– 4.00, 4.57]	19.42 (3.61)	19.33 (3.91)	– 0.09 (4.56) [– 4.65, 4.46]	0.48 [– 5.07, 6.04] 0.863	0.914
PMRQ R	18.52 (3.38)	19.71 (1.69)	1.19 (2.59) [– 1.40, 3.78]	20.0 (3.11)	17.76 (3.48)	– 2.23 (3.94) [– 6.18, 1.70]	– 1.0 [– 5.67, 3.67] 0.672	0.653
PMRQ TOT	38.0 (6.12)	39.57 (5.72)	1.47 (7.20) [– 5.73, 8.68]	39.28 (7.10)	37.23 (6.51)	– 2.04 (8.76) [– 10.8, 6.72]	– 0.21 [– 9.85, 9.43] 0.965	0.732

Data is presented as trimmed mean (*μ*_t_) and winsorized standard deviation (*σ*_w_). The effect size (ES_t_) corresponds to the difference between the two trimmed means (*μ*_t2 _− μ_t1_) in original units. The period effect was reported by a difference-in-differences (DID) analysis comparing differences in the outcome variables between the periods (DID = Δ2 − Δ1, where the difference in Period 1 is Δ_1_ and difference in Period 2 is Δ_2_) to report the period effect

*CI* confidence interval, *MoE*_*Δ*_ marge of error for the CI on the difference between the two trimmed means

Statistical significance (*p* < 0.05 of the two-tailed *p* value) for: *difference between groups in the period; ^†^period effect; ^‡^treatment effect

## Discussion

This double-blind randomized placebo-controlled crossover clinical trial aimed to evaluate the effects of a chewable NO_3_^−^-rich BR-based supplement on cognitive performance. We expected that the acute supplementation with a NO_3_^−^-rich product would have a positive impact on cognitive function in healthy individuals. We partially confirmed this initial hypothesis given that several, but not all neuropsychological tests showed significant difference between BR-CT and placebo conditions. Our results indicated a clinically significant improvement on both immediate (+ 20.69%) and delayed (+ 12.34%) memory capacity after BR-CT supplementation in comparison to the placebo condition. It is important to point out that the list of 15 words we administered with the RAVLT test was repeated five times, and at the testing time the participants had to repeat the words they remember. This process of reading the words five times put into action mechanisms of repetitive learning and memory consolidation at the short and long term that were enhanced by BR-CT supplementation. Notwithstanding, there were no significant changes (*p* > 0.05) on verbal and visuo-spatial short-term memory (digit span forward and backward). In addition, the lack of statistically significant variation on the PRMQ tests guarantees that there was no influence on the consciousness of carrying out a test. PRMQ is a self-report questionnaire to measure prospective and retrospective memory slips in everyday life. This test was properly included in the assessment to check the unconscious effects and the participants' perception with respect to any improvements in their performance. We should emphasize that the participants did not know when they would take the NO_3_^−^-rich BR-based supplement and when the placebo, and also that all were inexperienced in experimental research. Even though there was no significant improvement on information processing speed via SDMT, the results of the FAB test showed significant differences on the frontal lobe functions after BR-CT supplementation (+ 2.57%; *d*_unb_ = 0.314). The executive, abstraction and re-elaboration skills are characteristics of the frontal area and are also fundamental in the research processes of the most suitable strategies for memorization processes [66, 67]. Moreover, our neuropsychological measure of verbal fluency (COWAT/FAS) presented a clinically significant improvement after the administration of the BR-CT supplement (+ 11.16%; *d*_unb_ = 0.403). Finally, the other two tests concerning mood and anxiety revealed significant difference in BDI (*p* = 0.007), but not on STAI-Y1 and Y2 after BR-CT; nevertheless, the sequence and period effect that was found on STAI-Y2 makes clear that the condition might not have been a stable or certain between-subject variability for this test. Similar to other recent positive findings on cognitive reaction time and memory retrieval speed, our results showed that acute NO_3_^−^ supplementation improves certain areas of cognition. The improvements in frontal skills as well as lexical and memory capacity are elements that confirm an enhancement in general cognitive capacity. Complementarily, the BR-CT supplement used in this study was well tolerated (it did not show any kind of undesirable or adverse effect) and even the palatability was appreciated. This latter aspect should not be underestimated, not only for what concerns the compliance in use, but also this has allowed a longer stay in the mouth with consequent greater exposure to the salivary microbiota, with the consequent possible greater conversion of NO_3_^−^ into NO_2_^−^ and absorption in the plasma circulation. It might be possible that the increase in cerebral blood flow [5], and thereby the higher nutrient supply, after the supplementation with the chewable NO_3_^−^-rich BR-based product might have impacted the cognitive performance. Several studies highlight the NO synthesis in the brain and its role in various neuronal functions, including learning and memory processes, cortical arousal, nociception, food intake, penile erection, yawning, blood vessel dilation, and the immune response [68, 69]. Multiple neuronal mechanisms might be involved in how NO appears to affect learning, short-term memory, and long-term memory which is associated with changes in behavior while mammals are learning and at various post-learning periods [70]. Numerous studies have shown that short-term memory and long-term memory represent separate processes [71, 72]. In principle, NO transmission may or may not affect learning behavior long-term memory or all these processe, and this could also be in line with the studies of neuronal plasticity, which affirm that it is no longer just a single circuit that is responsible for specific cognitive functions. Indeed, the discovery that NO and H_2_S participate as second messengers that influence visual working memory will lead to a paradigm shift in our understanding of working memory mechanisms and the organizational features of brain structures [73]. In general, our collective findings revealed improvements on memory ability and frontal lobe functions in humans, albeit the reliance on NO activation of the computational ability of the brain [70] deserves further investigation. It is important to underline that the psychodiagnostic tests used in this study, and which investigate cognitive functions, consist of a parallel form. In the first phase, a specific form was administered and in phase 2 the same test was administered, with the same psychometric structure but a different form, so that the learning effect could be controlled. To be certain that the learning was not due to a memory of the elements already learned before and, therefore, to the test but to an effect of the integrator. However, it is essential to remember that the psychodiagnostic tests used in this study, which investigate cognitive functions, are of the parallel "structure form". In the first phase, a specific structure form was administered, and in the second phase, the test was with the same psychometric structure but a different type of form, to control the learning effect. To be certain, the learning was not due to a memory of the elements already learned before and, therefore, to the test but to an effect of the supplement. For example, the words of the RAVLT that the participant heard in the first administration were not the same as those heard in the second administration. Finally, although we did not find significant influence on the results based on the level of education, the intake of coffee (more than two espresso coffees per day—about 50–70 mg of caffeine) or the sex of participants, future studies might emphasize these associations and study individual responses. It needs to be noted that our crossover design avoided problems of comparability of BR-CT and placebo groups regarding confounding variables (e.g., age and sex).

### Limitations and strengths

The results of our study should be discussed in light of the following limitations and strengths. First, we did not measure the blood concentrations of NO_3_^−^, NO_2_^−^, or NO to ensure that there was a significant increase in this metabolite after BR-CT supplementation. Next, studies might evaluate if there is an actual and clinical difference between the chewable and drink versions of NO_3_^−^ supplementation on cognitive function and exercise performance. Secondly, we did not use near-infrared spectroscopy to detect changes in cerebral blood flow as an indirect measure of brain activity. Third, we did not measure NO_3_^−^ concentration in our BR-CT supplement to be sure the reported amount that has been relayed in literature for physiological benefits was given to the participants. However, RedNite^®^, (Enovate Biolife, Wilmington, DE) is a product with a range between 1.5 and 2.75% of standardized NO_3_^−^ concentration (equal to 45–82.5 mg of NO_3_^−^). Last, the wide range of the participants age that could lead difference cognitive performance and the not counterbalanced administration of the supplement. However, the following strengths must be noted. The double-blinding and the crossover design allowed participants to be exposed to both treatments in similar conditions, eliminated between-subject variability, and gave more statistical power because of paired comparisons [74]. In fact, each participant served as his/her own control, which distinguishes from a conventional parallel-group trial [57]. Moreover, a neuropsychological battery with validated and well-recognized tests to evaluate cognitive function was applied by a neuropsychologist with more than 10 years of experience.

## Future directions

Since NO_3_^−^ from vegetables, whether cooked or uncooked, is absorbed very effectively in healthy human participants (absolute nitrate bioavailability ≈ 100%) [75], upcoming research should evaluate the effects of high NO_3_^−^ diets on cognitive function for practical purposes. It is likely that there is an additive effect of dietary NO_3_^−^ following repeated consumption of BR; consequently, future research should study the chronic effects of the chewable versions of NO_3_^−^-rich products with the corresponding assessment of BR-dependent microbiota changes in different ages throughout the life span [76]. Finally, a recent randomized crossover clinical trial by Jackson et al. [80] demonstrated that BR juice co-supplementation with apple and coffee berry phenolic acid-rich extracts increased oxygen saturation in the frontal cortex and reduced mental fatigue [77]. Thus, future studies might focus on evaluating the effects of dietary nitrate in combination with other nutrients or bioactive compounds (e.g., phenolic-rich extracts).

## Conclusions

The acute administration of a chewable BR-based supplement improves certain aspects of cognitive function in healthy females and males, particularly, memory capacity and frontal skills. No significant changes were detected in both working memory and information processing speed after BR-CT supplementation. Although the chewable form of this BR-based supplement appeared to be safe and effective, more investigation in several population conditions is needed. The results of this study contribute to the body of evidence that focuses on the effects of NO_3_^−^ supplementation on cognitive performance.

## Data Availability

The data that support the findings of this study are available on request from the corresponding author, RC.
